# Pursuit of communal values in an agentic manner: a way to happiness?

**DOI:** 10.3389/fpsyg.2014.01320

**Published:** 2014-11-18

**Authors:** Andrea E. Abele

**Affiliations:** Social Psychology Group, University of Erlangen-NürnbergErlangen, Germany

**Keywords:** values, the big two, agency and communion, life satisfaction

## Abstract

The present research studies the association between traits, values, and life satisfaction. While values should influence the direction of an individual’s goals and behavior, his/her traits impact effort-expenditure, efficiency, and persistence in goal-pursuit. We apply the framework of the “Big Two” of agency and communion (Bakan, 1966) for distinguishing the content of values and traits. While agentic content refers to qualities relevant for goal-attainment, such as assertiveness, competence or persistence, communal content refers to qualities relevant for the establishment and maintenance of social relationships, such as being friendly, helpful, or fair. We predict that high scores on communal values and high scores on agentic traits are associated with life satisfaction. We test these predictions in two studies conducted in different countries (Germany and Russia) with different cultural background. The findings support our reasoning: across both countries we find positive associations of communal values and agentic traits with life satisfaction; and individuals high in communal values and high in agentic traits are most satisfied with their lives. In Russia, the association of communal values with life satisfaction is moderated by agentic traits; in Germany, however, there is a main effect of communal values.

## INTRODUCTION

There is a long research tradition on how people may achieve well-being and happiness, which is considered a major goal in life (Fredrickson, 2001; Gable and Haidt, 2005; Diener and Biswas-Diener, 2008; Diener, 2012). Individual differences approaches, for instance, show that extraversion and emotional stability are strongly associated with life satisfaction (Diener and Lucas, 1999). Social psychological approaches suggest that supportive social networks, having friends, and living with a spouse enhance life satisfaction (Argyle, 1999). Moreover, interactionist approaches study if the impact of situational conditions on an individual’s happiness varies with personality (for overviews see Diener and Biswas-Diener, 2008; Hefferon and Boniwell, 2011).

Life satisfaction is the cognitive component of an individual’s well-being, while positive and negative affect are the affective components. The present research adds a novel framework to the analysis of global life satisfaction, a cognitive appraisal of one’s life overall, which is distinct from domain-specific life satisfaction such as satisfaction with the self, with one’s social relationships, or one’s leisure time (Diener and Lucas, 1999; Diener, 2012).

The present approach introduces the agency/communion distinction into research on life satisfaction. The agency/communion distinction (a so called the “Fundamental Dimensions” or the “Big Two”; Paulhus and John, 1998; Abele and Wojciszke, 2014) has been influential in work on person perception, self-perception and personality, but not in research on life satisfaction. Additionally, we look at the joint impact of two variables that have so far seldom been studied in combination. These are an individual’s self-concept regarding agentic and communal traits and individuals’ agentic and communal values. We will first outline the basic concepts and present the theoretical reasoning. We will then test our hypotheses using data collected in two culturally different countries (i.e., Germany and Russia).

The distinction between agency and communion (A and C) is among the most influential pairings of content in psychology. Coined by Bakan (1966), these two conceptual labels have provided an effective framework for the analysis of traits, behaviors, values, motives, and social cognition (for reviews see Paulhus and John, 1998; Judd et al., 2005; Trapnell and Paulhus, 2012; Abele and Wojciszke, 2014). While agentic content refers to qualities relevant for goal-attainment, such as assertiveness, competence, or persistence (“getting ahead,” Hogan, 1982), communal content refers to qualities relevant for the establishment and maintenance of social relationships, such as being friendly, helpful, or fair (“getting along”; Hogan, 1982). Agency and communion constitute two separate clusters of meaning (Abele and Wojciszke, 2007). They capture the two recurring challenges of human life – pursuing individual goals and being a member of social groups and relationships (Ybarra et al., 2008). People may hold many different values and they may believe that they possess many different traits (i.e., concepts of the self). This variety is usefully categorized into a more limited number of classes of traits and values using as framework the A and C distinction.

Values are described as cognitive representations of basic motives or as rather stable broad life goals that are important to people in their lives and guide their perception, judgments, and behavior (Rokeach, 1973; Schwartz, 1992). They specify what is important in a culture and what is important for individuals (Trapnell and Paulhus, 2012). Values are motivational forces that influence goals and the direction of behavior.

The self-concept refers to an individual’s beliefs about himself or herself, including the person’s traits and who and what the self is (Baumeister, 1999). Beliefs about own traits (i.e., one component of the self-concept) should influence effort-expenditure and efficiency in behavior and goal-pursuit. If, for instance, a person believes himself/herself to be competent and assertive, then he/she may try harder and be more confident to reach his/her goals than if he/she believes not to be competent and/or assertive enough to be successful in goal-pursuit. A recent meta-analysis regarding the nature of the relationship between values and traits (Parks-Leduc et al., 2014) revealed moderate associations demonstrating that traits and values are distinct constructs.

In this context, a rich set of data shows that both values (Trapnell and Paulhus, 2012) and people’s spontaneous self-descriptions (Diehl et al., 2004; Uchronski, 2008) can be organized into the A and C framework; thus, giving a clearer picture of the link and differences between values and the self-concept. People, for example, differ in the importance they place on “getting ahead” (A values) versus “getting along” (C values). These basic values are, in turn, deeply connected to personality and an individual’s self-concept, as they emerge in the socialization process. Indeed, self-ratings on various trait scales also result in these two content factors (A & C; Abele and Wojciszke, 2007, 2014). People describe themselves with different degrees of agentic and communal traits.

In regard to values, most cultures hold C values in higher regard than A values (Trapnell and Paulhus, 2012). Acting in accord to the values in one’s culture fosters an individual’s well-being (Myers and Diener, 1995). Furthermore, self-determination theory (Kasser and Ryan, 1996) distinguishes between “intrinsic” goals like affiliation and feelings of community and “extrinsic” goals like aspirations for financial success and social recognition. Albeit intrinsic and extrinsic goals are not the same as C and A values they nevertheless are related. Intrinsic goals are related to C values, and extrinsic goals are related to A values. Self-determination research shows that intrinsic goals are associated with well-being, whereas extrinsic goals are associated with lower vitality (e.g., Kasser and Ryan, 1996; Kasser et al., 2014). Hence, both the higher appreciation of C values in many cultures and research on the pursuit of intrinsic versus extrinsic goals suggests that C values are more strongly related to life satisfaction than A values. In support to this prediction Hofer et al. (2006) found that values in the domain of intimacy-affiliation (the C domain in the present terminology) were associated with life satisfaction. Values in the domain of power (the A domain in the present terminology), however, were independent of life satisfaction. An indirect evidence for a positive association between C values and life satisfaction comes from studies on spending or helping others. These behaviors – which are mainly based on C values – enhance an individual’s well-being (Dunn et al., 2012). Research showing a positive association between social relationships and life satisfaction (Argyle, 1999; Diener and Biswas-Diener, 2008) does also indirectly suggest that C values may be more closely related to life satisfaction than A values.

On the other hand, research on A and C traits shows a different pattern. For example, research by Helgeson (1994) showed that A traits are associated with reduced depression, anxiety, and health complaints; whereas C traits show less clear associations with well-being parameters. Similarly, Saragovi et al. (2002) found that although A traits and C traits were both positively related to positive affect and social adjustment, only A traits were positively associated with life satisfaction. In addition, A traits are correlated with variables that are also correlated with life satisfaction (Çivitci and Çivitci, 2009; Kong et al., 2014), such as, self-esteem (Abele et al., 2008a; Wojciszke et al., 2011; Gebauer et al., 2013) and self-efficacy beliefs (Abele, 2003). In contrast to C traits, studies show that A traits predict longitudinal success in an individual’s occupational career (Kirchmeyer, 1998; Abele, 2003; Abele and Spurk, 2011), that A traits are also malleable in response to success and failure experiences (Abele et al., 2008a; see also Uchronski et al., 2012), and that A traits predict an individual’s feelings of competence (Locke and Nekich, 2000; see also Locke, 2003). In sum, A traits seem to enhance an individual’s feelings of competence and of self-efficacy; A traits are positively associated with self-esteem; and A traits are related to reduced depression and health complaints. Moreover, A traits instigate behavior which is success-oriented and leads to actual to success. C traits do not show this pattern of associations. As feelings of competence, of self-efficacy, positive self-esteem, and success experiences are associated with life satisfaction, thus, also suggesting that A traits are associated with life satisfaction both directly but also indirectly.

## THE PRESENT STUDY

An individual’s values and his/her beliefs about own traits may be organized into the A and C framework. With this framework in mind, earlier research points to an asymmetry, suggesting that C values are more beneficial for an individual’s life satisfaction than A values (Kasser and Ryan, 1996; Locke, 2003; Hofer et al., 2006; Dunn et al., 2012; Kasser et al., 2014) and that A traits are more beneficial for an individual’s life satisfaction than C traits (Helgeson, 1994; Saragovi et al., 2002). We therefore predict that people with high C values and with high A traits are more satisfied with their lives than people with low C values and low A traits. Also in this line, if an individual feels that he/she does not have the competence and persistence (i.e., high in A traits) to follow her C values then the effect of values on life satisfaction may become smaller. In other words, we will also investigate if the association of C values with life satisfaction is moderated by A traits.

As there are cultural differences in the determinants of life satisfaction (Oishi et al., 1999; Diener and Lucas, 2000), our hypotheses will be studied in two different cultures, Germany and Russia. Germany and Russia differ in economic wealth and in several important culture dimensions (http://geert-hofstede.com/germany.html; Hofstede, 2001). Germany is, for example, a more individualistic and masculine culture than Russia (Hofstede, 2001). Nevertheless, data suggests that the stronger association of A traits, compared to that of C traits, with self-esteem does not only hold in individualistic cultures like Germany (Abele et al., 2008a; Gebauer et al., 2013), but also in somewhat more collectivistic cultures like Poland (Wojciszke et al., 2011) and even in a clearly collectivistic culture like China (Bi et al., 2013). In specific samples of Southern China, however, researchers also found a positive association of C traits with self-esteem, but this was lower than the one between A traits and self-esteem (Bi et al., 2013). It seems that the stronger association of A traits with self-esteem than of C traits with self-esteem holds across cultures. In addition, Hofer et al. (2006) found that the positive association between C values and life satisfaction did not differ between cultures (Cameroon, Costa Rica, Germany); and the zero association between A values and life satisfaction also holds across these cultures. Even if this set of findings are still small, it suggests that the association between values, traits, and life satisfaction may be similar across cultures.

### HYPOTHESES

To sum up, we state the following hypotheses: (1) C values are more strongly related to life satisfaction than A values. (2) A traits are more strongly related to life satisfaction than C traits. Moreover, we will test if the relation between C values and life satisfaction is moderated by A traits.

## STUDY 1: GERMANY

### PARTICIPANTS

We recruited 201 participants (128 women, 73 men; age range from 15 to 72 years; *M* = 27.36, SD = 12.94). Most of them had graduated from university (79%). They filled out an online questionnaire.

### MATERIALS AND MEASURES

A and C values were assessed with a scale constructed by Trapnell and Paulhus (2012). Participants rated the importance of 20 values on a 7-point scale ranging from 1 = *not important for me* to 7 = *highly important for me*. A values were “autonomy,” “competence,” “achievement,” “ambition,” “influence,” “power,” “status,” “wealth,” “recognition,” and “superiority.” C values were “trust,” “honesty,” “harmony,” “civility,” “loyalty,” “politeness,” “compassion,” “altruism,” “forgiveness,” and “equality.” The reliabilities of both scales were good (A values, α = 0.85; C values, α = 0.88).

A and C traits were measured by means of 40 bipolar adjective scales (Abele and Hauke, unpublished manuscript). Answers were given on bipolar scales ranging from 2 (*definitely applies to me*) for the left-hand adjective (for instance “efficient”) through 0 (*neither – nor*) to 2 (*definitely applies to me*) for the right-hand adjective (for instance “inefficient”). These ratings were later recoded into 5-point scales with 5 being the positive endpoint of the scale (for instance 1 “inefficient” to 5 “efficient”). Further examples for the A scale are “competent” versus “incompetent” and “gives up easily” versus “does not give up easily.” Examples for the C scale are “helpful” versus “not helpful” and “fair” versus “unfair.” The reliability of both scales was good (A traits, α = 0.87; C traits, α = 0.89).

We measured life satisfaction with a German version of the satisfaction with life scale (Diener et al., 1985; German version Glaesmer et al., 2011). It comprises five items (sample item: “In most ways, my life is close to my ideals”; “So far I have gotten the things I want in my life”) which were answered on a 5-point scale from 1 = *not at all* to 5 = *definitely agree*. The reliability of the scale was good (α = 0.82).

### RESULTS AND DISCUSSION

We first conducted a series of confirmatory factor analyses (CFAs) using MPlus (Muthén and Muthén, 1998) to ensure the distinctness of the values and the trait scales. We followed suggestions by Little et al. (2002) and used item parcels for these analyses. Item parcels have better psychometric characteristics than single items and fewer parameters are needed to define a construct. Six item parcels were built for the agency and communion trait measures each. Three parcels were built for the A and C value measures each. Using a maximum likelihood estimation method, the results of the CFA revealed that the four-factor model that distinguishes between A and C values and A and C traits fitted the data [χ^2^ = 259.20, *df* = 125, *p* < 0.001; comparative fit index (CFI) = 0.90, Tucker-Lewis Index (TLI) = 0.88, root mean square error approximation (RMSEA) = 0.07, standardized root mean square residual (SRMR) = 0.07; cf., Hu and Bentler, 1999]. Most importantly, this model provided a significant improvement in fit compared to a model with only two factors (A values plus traits versus C values plus traits; Δχ^2^ = 155.96, *df* = 5, *p* < 0.001)^1^. We therefore used the four scales of A and C values and of A and C traits.

**Table 1** shows the means, standard deviations and inter-correlations of the present measures. Women endorsed C values slightly more than men; A traits were positively correlated with age; and participants with higher education scored lower on C traits than participants with lower education. Moreover, older people endorsed A values less than younger ones.

**Table 1:** Mean, standard deviation, and intercorrelation (Study 1; *N* = 201).

			Correlation with						
	*M*	*SD*	2	3	4	5	6	7	8
(1) Gender^a^			0.11	–0.04	0.03	–0.12	0.07	–0.15*	–0.07
(2) Age	27.36	12.94		–0.11	0.18*	0.01	–0.15*	–0.08	0.03
(3) Education^b^					0.02	–0.16*	0.12	–0.09	0.11
(4) Agentic traits^c^	3.63	0.50				0.33***	0.39***	0.16*	0.40***
(5) Communal traits^c^	4.06	0.48					–0.03	0.68***	0.20**
(6) Agentic values^d^	4.31	0.99						0.05	0.15*
(7) Communal values^d^	5.77	0.86							0.21**
(8) Life satisfaction^c^	3.68	0.81							

^a^0 woman, 1 man; ^b^0 lower than high school, 1 high school and more; ^c^scale from 1 to 5; ^d^scale from 1 to 7; *p < 0.05; **p < 0.01; ***p < 0.001.

Participants rated their A traits lower than their C traits, *t*(200) = 10.83, *p* < 0.001, *d* = 0.78, and they endorsed A values less than C values, *t*(200) = 16.25, *p* < 0.001, *d* = 1.15. A traits and C traits were significantly correlated, but A values and C values were independent. Life satisfaction was significantly correlated with both the value scales and the trait scales.

In order to test the above hypotheses, we ran a stepwise multiple regression (all variables centered). We first regressed the socio-demographics, then the value scales, then the trait scales and in the fourth step the interaction between A traits and C values (Aiken and West, 1991) on life satisfaction.

The findings are depicted in **Table 2**. Gender, age, and education had no influence on life satisfaction. Supporting H1, C values were significantly associated with life satisfaction, but A values were not. Supporting H2, A traits related positively to life satisfaction and C traits showed no association^2^. The exploratory test for an interaction of C values and A traits revealed no effect.

**Table 2:** Socio-demographic variables, values, and self-concept regressed on life satisfaction (Study 1; *N* = 201).

	First step β, *SE*	Second step β, *SE*	Third step β, *SE*	Fourth step β, *SE*	Final model overall adjusted R^2^
Gender	–0.07 (0.12)	–0.05 (0.12)	–0.05 (0.11)	–0.05 (0.11)	
Age	0.05 (0.01)	0.08 (0.01)	–0.02 (0.01)	–0.01 (0.00)	
Education	0.11 (0.14)	0.12 (0.14)	0.11 (0.13)	0.11 (0.13)	
Agentic values		0.14 (0.06)	–0.03 (0.06)	–0.01 (0.06)	
Communal values		0.21*** (0.07)	0.18* (0.09)	0.19* (0.09)	
Agentic traits			0.40*** (0.13)	0.40*** (0.13)	
Communal traits			–0.05 (0.16)	–0.02 (0.17)	
A traits * C values				0.10 (0.05)	0.17***

Δ R^2^	0.00	0.06**	0.12***	0.01	

*p < 0.05; **p < 0.01; ***p < 0.001.

Summarizing, Study 1 revealed findings in support of our hypotheses. Participants who endorsed C values (H1) and who rated their A traits as high (H2) were especially satisfied with their lives. Our exploratory test for a moderation of the effects of C values via A traits revealed no effect.

Moreover, participants rated their C traits higher than their A traits. This is the usual finding in the literature (see Abele and Wojciszke, 2014, for an overview). Also in support of findings in the literature (Trapnell and Paulhus, 2012), they endorsed C values more than A values. It may be asked why older participants rated their A traits higher than younger ones. We think that this is due to the sample as we had only few participants above the age of 50 (*N* = 13), i.e., the “older” participants were “middle-aged.” There are also findings in the literature suggesting a positive association between persistence (belonging to the A domain) and age (Josefsson et al., 2013).

## STUDY 2: RUSSIA

### PARTICIPANTS AND MEASURES

We recruited 328 participants (213 women, 115 men; age range from 15 to 66 years; *M* = 27.93, SD = 9.34). Most of them had graduated from university (87%). They filled out an online questionnaire. The measures were the same as in the first study. The reliabilities of the scales were good (A traits, α = 0.88; C traits, α = 0.83; A values, α = 0.87; C values, α = 0.86; life satisfaction, α = 0.81).

### RESULTS AND DISCUSSION

We again conducted a series of CFAs with the analogous item parcels as in Study 1. The four-factor model (A and C values and A and C traits) fitted the data adequately (χ^2^ = 424.64, *df* = 125, *p* < 0.001; CFI = 0.89, TLI = 0.86, RMSEA = 0.09, SRMR = 0.08). This model provided a significant improvement in fit compared to a model with only two factors (A values plus traits versus C values plus traits; Δχ^2^ = 529.25, *df* = 5, *p* < 0.001)^3^. We therefore again used the four scales of A and C values and of A and C traits.

**Table 3** shows the mean, standard deviation, and inter-correlation. Women scored higher on C traits and higher on C values than men; women were also more satisfied with their lives than men. Older people scored higher on A traits, they were more satisfied with their lives, and they endorsed A values less than younger people. People with higher education scored higher on A traits than people with lower education.

**Table 3:** Mean, standard deviation, and intercorrelation (Study 2; *N* = 328).

			Correlation with						
	*M*	*SD*	2	3	4	5	6	7	8
(1) Gender^a^			–0.07	–0.09	0.07	–0.13**	–0.07	–0.21***	–0.12*
(2) Age	27.93	9.34		0.21***	0.14**	0.06	–0.25***	0.11*	0.20***
(3) Education^b^					0.11*	0.08	0.00	.08	0.05
(4) Agentic traits^c^	3.57	0.52				0.40***	0.30***	0.18***	0.36***
(5) Communal traits^c^	4.01	0.42					0.09	0.57***	0.35***
(6) Agentic values^d^	4.70	1.06						0.17**	0.06
(7) Communal values^d^	5.68	0.86							0.22***
(8) Life satisfaction^c^	2.98	0.87							

^a^0 woman, 1 man; ^b^0 lower than high School, 1 high school and more; ^c^scale from 1 to 5; ^d^scale from 1 to 7; *p < 0.05; **p < 0.01; ***p < 0.001.

Participants again scored lower on A traits than on C traits, *t*(327) = 15.19, *p* < 0.001, *d* = 0.86, and they endorsed A values less than C values, *t*(327) = 14.20, *p* < 0.001, *d* = 0.72. A traits and C traits were significantly correlated and A values and C values were also slightly correlated. Life satisfaction was significantly correlated with both A traits and C traits and with C values, but not with A values.

**FIGURE 1:**
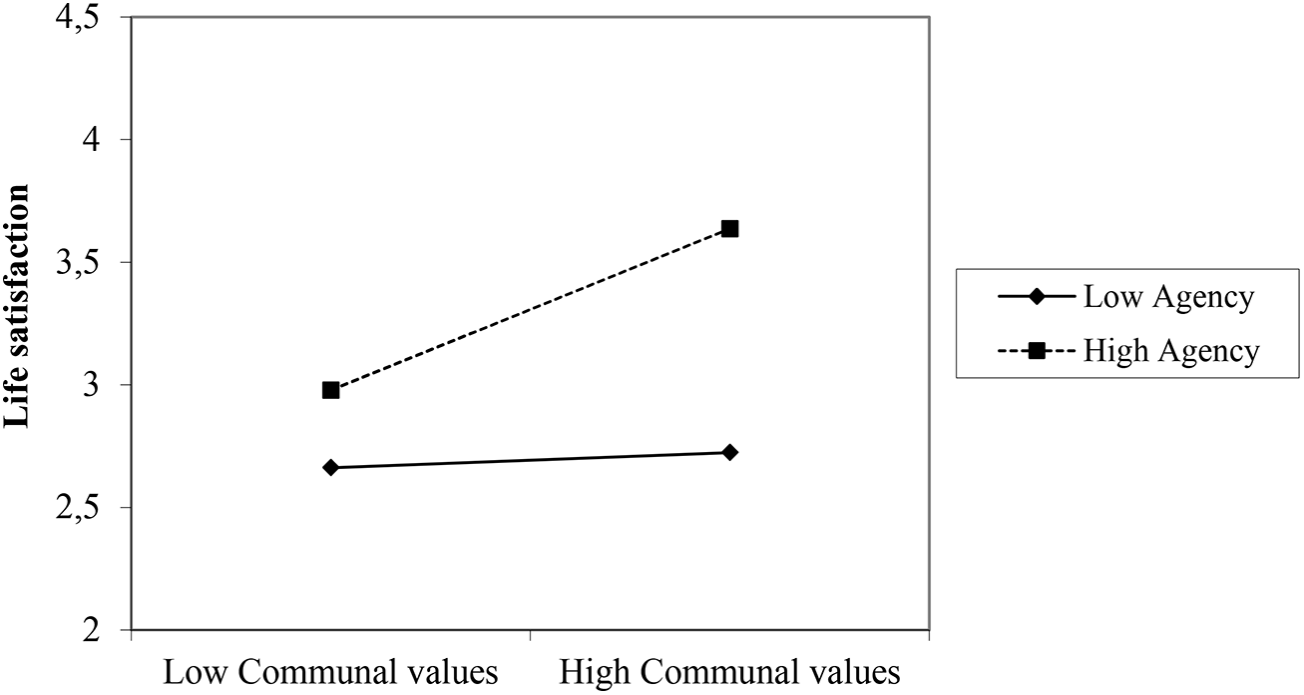
**Interaction of C values and A Traits on life satisfaction (Study 2; Russia; Low: ≤one SD below mean; High: ≥one SD above mean)**.

The hypotheses were again tested by means of a stepwise multiple regression. The variables were centered before entering them into the regression (**Table 4**). In the Russian sample women and older people were more satisfied with their lives than men and younger people. Supporting H1, C values were significantly associated with life satisfaction (second step). However, the influence of C values disappeared, when the trait measures were introduced in step three. Now both A and C traits significantly related to life satisfaction^4^. Step four, finally, revealed a significant interaction of A traits and C values. As can be seen in **Figure 1**, the impact of C values on life satisfaction was moderated by A traits. Participants high in A traits were generally more satisfied than those low in A traits. C values had an impact on life satisfaction especially when participants were high in A traits.

**Table 4:** Socio-demographic variables, values, and self-concept regressed on life satisfaction (Study 2; *N* = 328).

	First step β, *SE*	Second step β, *SE*	Third step β, *SE*	Fourth step β, *SE*	Final model Overall adjusted R^2^
Gender Age Education	–0.11* (0.10) 0.19** (0.01) 0.00 (0.14)	–0.07 (0.10) 0.20** (0.01) –0.01 (0.14)	–0.11* (0.09) 0.14** (0.01) –0.04 (0.13)	–0.12* (0.09) 0.13* (0.01) –0.06 (0.13)	
Agentic values Communal values		0.08 (0.05) 0.18*** (0.05)	–0.02 (0.05) 0.02 (0.06)	–0.01 (0.05) 0.07 (0.06)	
Agentic traits Communal traits			0.27*** (0.10) 0.21** (0.14)	0.30*** (0.10) 0.19** (0.13)	
A traits * C values				0.18*** (0.04)	0.23***

Δ R^2^	0.05**	0.04**	0.13***	0.03*	

*p < 0.05; **p < 0.01; ***p < 0.001.

Summarizing, H1 was again supported: C values were positively associated with life satisfaction. However, as the significant interaction with A traits showed this effect was moderated by A traits. C values were more closely associated with life satisfaction when A traits were high. Supporting H2, A traits were generally positively associated with life satisfaction.

Replicating Study 1, participants rated their C traits higher than their A traits; and they endorsed C values more than A values. Also replicating Study 1, older people rated their A traits higher than younger people. However, there were again only few people above the age of 50 (*N* = 9). Whereas the socio-demographic variables had no influence in the German sample, the Russian sample revealed higher life satisfaction of women and older people.

## COMPARISON BETWEEN BOTH STUDIES

We compared the trait-, value-, and life satisfaction measures between both samples. Russians and Germany did not differ in their trait ratings [A traits: *t*(527) = 1.26, *ns*; C traits: *t*(527) = 1.29, *ns*] as well as in C values [*t*(527) = 1.23, *ns*]. However, life satisfaction was higher in the German sample, *t*(527) = 9.15, *p* < 0.001; *d* = 0.82; and A values were higher in the Russian sample, *t*(527) = 4.26, *p* < 0.001; *d* = 0.38^5^.

We also compared the correlations between values and traits across the studies. A values and A traits correlate significantly (Germany: *r* = 0.39, *p* < 0.001; Russia: *r* = 0.30, *p* < 0.001), but C values and C traits correlate even more (Germany: *r* = 0.68, *p* < 0.001; Russia: *r* = 0.57, *p* < 0.001).

**FIGURE 2:**
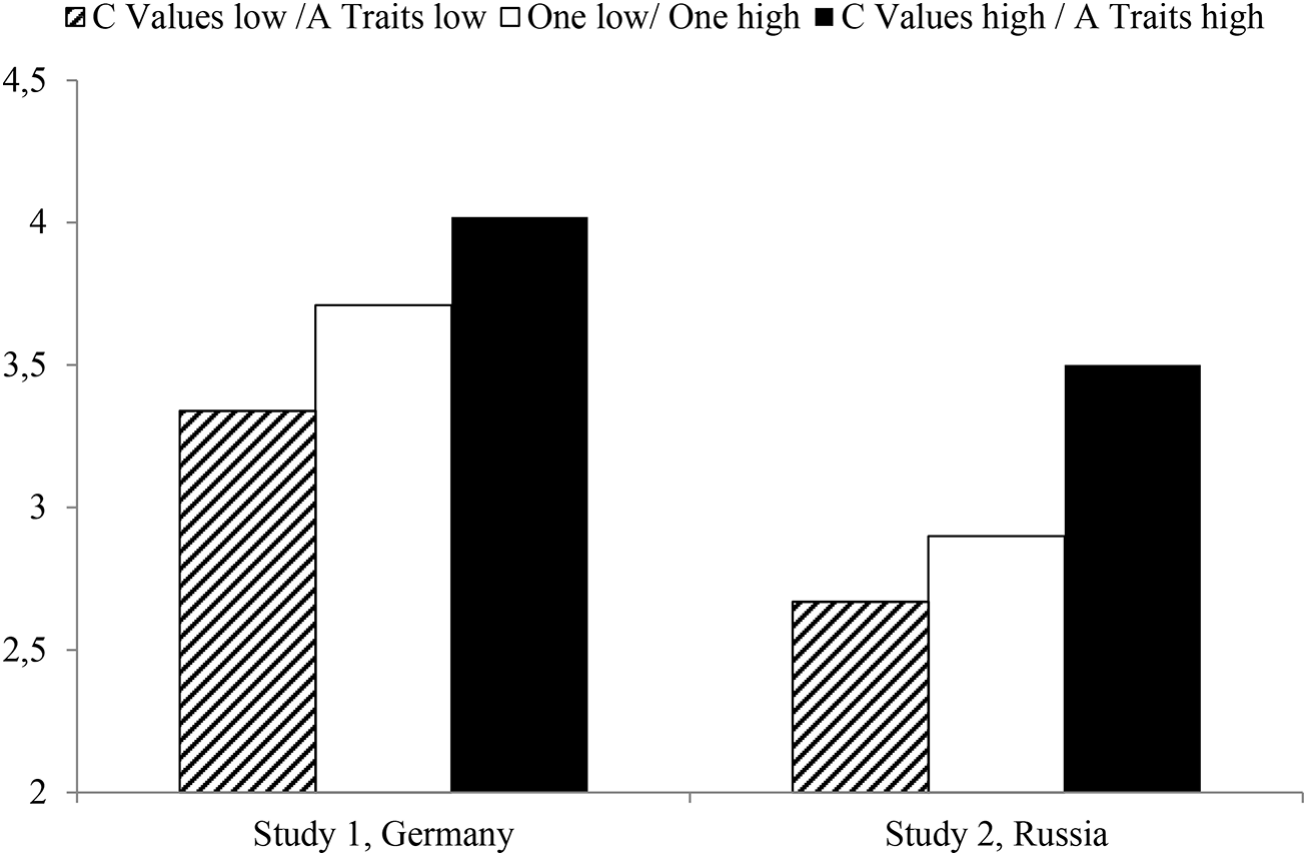
**Life satisfaction in Study 1 (Germany) and Study 2 (Russia) dependent on below versus above Median C Values and A traits**.

**Figure 2**, finally, summarizes the findings for our main hypotheses. In both samples we analyzed life satisfaction dependent on the individuals’ below versus above median A traits and dependent on their below versus above median C values. As **Figure 2** shows, the high/high group was always more satisfied than the middle group, which was more satisfied than the low/low group. The comparisons between these groups were highly significant^6^.

## DISCUSSION

### GENERAL FINDINGS

The present research studies the joint impact of values and traits on an individual’s life satisfaction. We have argued that values as motivational forces influence the direction of behavior and goals; and that traits should influence the effort and efficiency with which an individual strives for certain values and pursues certain goals. We applied the A & C framework to the analysis of values and traits and distinguished between A and C values (Trapnell and Paulhus, 2012) and between A and C traits (Abele and Wojciszke, 2007, 2014). We predicted that C values and A traits would be related to life satisfaction. We also tested if the association between C values and life satisfaction is moderated by A traits. The hypotheses were supported in the German sample and they were mainly supported in the Russian sample.

Supporting H1, C values were in both samples positively associated with life satisfaction. Whereas this was a general influence in the German sample, in the Russian sample the impact of C values was moderated by A traits. These findings are in line with our theoretical reasoning. C values add to life satisfaction once, because they are regarded as more important across cultures (in both samples C values were more endorsed than A values); and also because they are “intrinsically” rewarding (Kasser and Ryan, 1996; Kasser et al., 2014). A values had no influence on life satisfaction, and this finding was the same across both studies (cf. Hofer et al., 2006; Dunn et al., 2012). It is again in line with self-determination theory as A values are more related to “extrinsic” goals (Kasser and Ryan, 1996; Kasser et al., 2014). Moreover, A values are culturally less appreciated than C values (Myers and Diener, 1995).

Supporting H2, A traits were significantly associated with life satisfaction in both samples. Individuals high in A traits are self-confident and efficient – whatever goal or value they strive for. They are success-oriented and successful. In this context, self-confidence, self-efficacy, and self-esteem as well as success enhance a person’s life satisfaction (Helgeson, 1994; Abele et al., 2008a; Hefferon and Boniwell, 2011; Bi et al., 2013).

As was shown in **Figure 2**, individuals with above median C values and above median A traits were always more satisfied than individuals in the middle group (either C values or A traits below median) than those in the low/low group (individuals with below median C values and below median A traits).

The impact of A traits on life satisfaction was higher than the impact of C values; suggesting that not values *per se* impact a person’s life satisfaction, but the transformation of values into behavior is important. Consequently, beliefs in one’s own competence and assertiveness, that is, A traits, foster the transformation of values into behavior. Most importantly, the present data showed that the efficient pursuit of goals enhances life satisfaction when these goals are related to C values, such as, trust, honesty, altruism, forgiveness, and equality^7^. These findings are an addition to earlier research in which either A traits or the endorsement of C values was related to life satisfaction (e.g., Helgeson, 1994; Kasser and Ryan, 1996; Saragovi et al., 2002; Locke, 2003; Hofer et al., 2006; Dunn et al., 2012; Kasser et al., 2014).

The relatively strong association of A traits with life satisfaction was shown in both Germany and Russia. This intercultural finding supports our theoretical reasoning that a person’s agency being indicative of competence- and efficiency beliefs enhances effort-expenditure and optimism in goal striving and that these factors add to a person’s life satisfaction. The present research also adds to our understanding of the A & C framework in personality and social psychology. The data show that C traits and C values were more endorsed than A traits and A values. These findings are in line with prior reasoning and data on the primacy of communion (Abele and Bruckmüller, 2011; Abele and Wojciszke, 2014).

Moreover, the present results show that C values and C traits are more strongly correlated than A values and A traits; thus, supporting a “primacy of communion” not only in ratings of the self and of others, but also at the construct level. Communal content is more similar across languages than agentic content (Abele et al., 2008b; Abele and Bruckmüller, 2011; Abele and Wojciszke, 2014) and within a language it is more closely connected (“denser”) than agentic content (Bruckmüller and Abele, 2013).

### COMPARISON BETWEEN BOTH COUNTRIES

In contrast to the German sample, in which C traits showed no association to life satisfaction when A traits were controlled for, C traits were associated with life satisfaction in the Russian sample even when A traits were controlled for. As collectivism and femininity are more pronounced in the Russian culture than in the German culture (Hofstede, 2001), an individual’s C traits in the sense of warmth, friendliness and helpfulness might add more to life satisfaction in Russia than in Germany (for differences within China; Bi et al., 2013).

There are also both similarities and differences between the countries. Whereas in both samples women endorsed C values more than men, there were no further gender differences in the German sample but two more ones in the Russian sample. In the Russian sample women scored higher on C traits than men. This may be due to more traditional gender roles in Russia than in Germany (Fuwa, 2013). Russian women were also more satisfied with their lives than Russian men. However, gender did not moderated the association between self-concept, values and life satisfaction.

In both samples older participants rated their A traits higher than younger ones, and in both samples participants with higher education scored higher on A traits than those with lower education. These findings, however, should be replicated as there were only few older participants and only few less well educated participants in both samples.

It is not astonishing that life satisfaction of the German sample was higher than life satisfaction of the Russian sample, as Germany is a wealthier and safer country than Russia. It is more astonishing that A values were higher in the Russian sample than in the German one – given that Russia was classified as a less masculine country than Germany (Hofstede, 2001; http://geert-hofstede.com/germany.html) and that masculinity and A values share some common meaning (Abele and Wojciszke, 2007). One interpretation may be that the daily struggle for better living conditions is harder in Russia and that this experience leads to higher A values. Given the classification of Russia as less masculine and more collectivistic than Germany it is also astonishing that both samples did not differ in their trait ratings.

### LIMITATIONS

There are some limitations of the present research which should be addressed in future studies. First, our samples comprised mainly highly educated individuals. Albeit the present data did not show a moderating impact of level of education, the samples comprised nevertheless mainly highly educated individuals. The impact of education should be further studied. Second, our data are cross-sectional and it has to be demonstrated if A traits and C values influence life satisfaction in a longitudinal perspective. We are, however, confident that the present data do have some validity as they were replicated across two different countries. Third, it may be argued that Russia and Germany do not differ so much and that a better test of the cultural invariance of the present findings would be to involve countries which are more different like, for instance, China or Japan. Again, this is an issue for further research. Finally, it might be argued that the measures of A and C values as well as A and C traits were highly correlated and it is not clear if they really measure different constructs. We think that even though the measures were correlated we showed that a four-factorial model covers the data better than a two-factorial model (or any three-factorial model) and we also showed that values and traits are differentially related to life satisfaction. Hence, suggesting that the constructs of A and C values and A and C traits are sufficiently distinct (see also Parks-Leduc et al., 2014) even though the model fits in the CFA’s were not fully satisfactory.

## CONCLUSION

The present research adds to our understanding of individual differences underlying differences in life satisfaction. Values as the cognitive representation of motives are important. However, not all kind of values add to life satisfaction: in both present samples C values add to life satisfaction especially when people are convinced that they are “agentic” enough to pursue and live these values.

## Conflict of Interest Statement

The author declares that the research was conducted in the absence of any commercial or financial relationships that could be construed as a potential conflict of interest.

## Footnotes

1 We also tested several three-factor models, but they showed worse fit indices than the four-factor model (all *p*s < 0.001).

2 The regression of socio-demographics and self-concept (without values) revealed again a highly significant beta weight for A traits (β = 0.38, *p* < 0.0001), but not for C traits (β = 0.08, *ns*).

3 We also tested several three-factor models, but they showed worse fit indices than the four-factor model (all *p*s < 0.001).

4 The regression of socio-demographics and self-concept (without values) revealed highly significant beta weights for both A traits (β = 0.27, *p* < 0.001) and C traits (β = 0.22, *p* < 0.001).

5 Russians scored higher on eight of the A values (autonomy, competence, achievement, wealth, recognition, status, power, and superiority). There was no difference regarding the value of influence. Conversely, Germans scored higher on ambition.

6 Overall *F*-test: Germany, *F*(2,198) = 11.11, *p* < 0.001; Russia, *F*(2,325) = 26.14, *p* < 0.001. Duncan-tests between groups always *p* < 0.05. Difference high/high versus low/low in Germany: Cohen’s *d* = 0.93; in Russia: Cohen’s *d* = 1.04.

7 It might be argued that the effective pursuit of any value enhances life satisfaction. We therefore additionally tested if the interaction of A traits and A values is significant. However, in both samples, there was no interaction.
